# Ionic current correlations are ubiquitous across phyla

**DOI:** 10.1038/s41598-018-38405-6

**Published:** 2019-02-08

**Authors:** Trinh Tran, Cagri T. Unal, Daniel Severin, Laszlo Zaborszky, Horacio G. Rotstein, Alfredo Kirkwood, Jorge Golowasch

**Affiliations:** 1 Federated Department of Biological Sciences, New Jersey Institute of Technology, University Heights, Newark, NJ 07102 USA; 2 Johns Hopkins Zanvyl Krieger Mind/Brain Institute, Rm 350 Dunning Hall, and The Solomon H. Snyder Department of Neuroscience Johns Hopkins University, 3400 N. Charles St., Baltimore, MD 21218 USA; 3 Center for Molecular and Behavioral Neuroscience, Behavioral and Neural Science Graduate Program, Rutgers University-Newark, Newark, NJ 07102 USA; 4 Institute for Brain and Neuroscience Research, New Jersey Institute of Technology, University Heights, Newark, NJ 07102 USA; 5 Present Address: TED University, Department of Psychology. Ziya Gokalp Caddesi No. 48 06420, Kolej Cankaya, Ankara, Turkey; 6 Corresponding Investigator, CONICET, Argentina

## Abstract

Ionic currents, whether measured as conductance amplitude or as ion channel transcript numbers, can vary many-fold within a population of identified neurons. In invertebrate neuronal types multiple currents can be seen to vary while at the same time their magnitudes are correlated. These conductance amplitude correlations are thought to reflect a tight homeostasis of cellular excitability that enhances the robustness and stability of neuronal activity over long stretches of time. Although such ionic conductance correlations are well documented in invertebrates, they have not been reported in vertebrates. Here we demonstrate with two examples, identified mouse hippocampal granule cells (GCs) and cholinergic basal forebrain neurons, that the correlation of ionic conductance amplitudes between different ionic currents also exists in vertebrates, and we argue that it is a ubiquitous phenomenon expressed by many species across phyla. We further demonstrate that in dentate gyrus GCs these conductance correlations are likely regulated in a circadian manner. This is reminiscent of the known conductance regulation by neuromodulators in crustaceans. However, in GCs we observe a more nuanced regulation, where for some conductance pairs the correlations are completely eliminated while for others the correlation is quantitatively modified but not obliterated.

## Introduction

Ionic current levels in populations of identical neurons are extremely variable^1–10^. This poses the question of how neurons of a given type manage to generate consistent activity patterns despite the sometimes enormous variability (several fold) of the currents they express. One mechanism that has been proposed is the co-regulated expression of ionic channels^11–13^, which is revealed as correlations of conductances or transcript numbers in populations of identical cells^13,14^. The correlated expression of ionic currents, maximal conductances and ion channel transcript levels among populations of identical neurons have been observed in several neuronal cell types of invertebrate species^12,15–18^. However, that has typically been assumed to be an invertebrate idiosyncrasy. Evidence of their existence in vertebrates has been largely anecdotal or indirect^6,19,20^, and the only existing report of current correlations in vertebrates shows a correlation of voltage dependence and kinetic parameters^19,21^, but not the type of correlations described above. Nevertheless, there is ample theoretical work that suggests that ionic current amplitude correlations allow neurons of any type or species to express similar patterns of activity despite expressing widely different ionic current amplitudes by maintaining constant the relative levels of different current types^3,22–26^. Added to this, there is evidence that the expression of ionic current correlations is a highly regulated phenomenon^15^, suggesting that correlations play important roles in the long-term dynamics of neuronal activity, in the regulation of the robustness of this activity, or both.

Here we test the hypothesis that ionic current correlations are widely distributed across animal species, and demonstrate that ionic current amplitude correlations are also expressed in mammalian neurons. We argue that this is a ubiquitous phenomenon observed in species across phyla. We further show evidence that suggests that these correlations in mammalian neurons are regulated in a circadian-like manner.

## Results

### Hippocampal granule cells (GCs)

**Figure 1:**
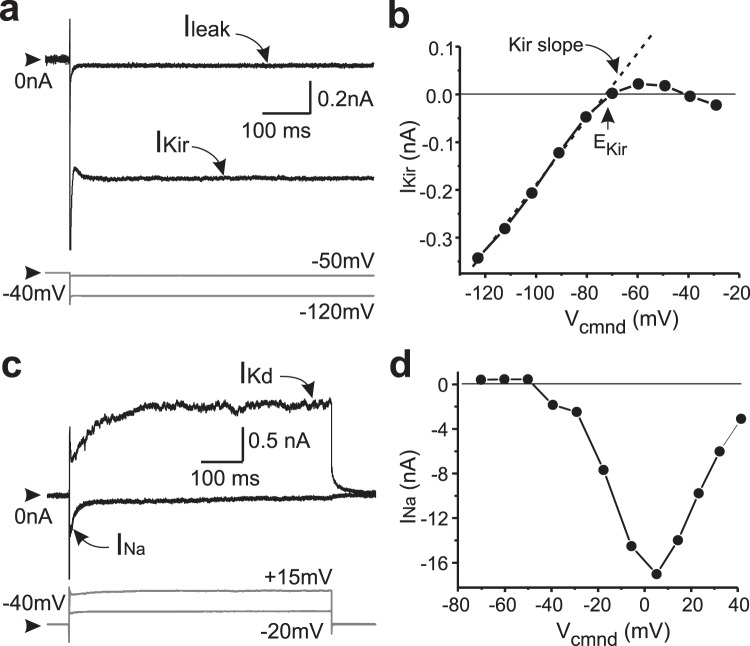
Several ionic currents can simultaneously be measured in mouse hippocampal dentate gyrus (DG) granule cells (GCs). I_Kd_, I_Na_, I_Kir_ and I_leak_ were measured with whole cell patch clamp in identified GCs. (**a**) Typical current traces from which inward rectifier (I_Kir_) and leak conductances were derived. (**b**) Sample I-V curve for leak-subtracted I_Kir_ showing E_Kir_ and g_Kir_ (slope) measurements. (**c**) Examples of delayed rectifier (I_Kd_) and early TTX-sensitive inward current (I_Na_). (**d**) Sample I-V curve of TTX-sensitive Na current. In a and c, top traces show currents; bottom (gray) traces show the pipette potentials at which the currents were measured (−50 mV for I_leak_, −120 mV for I_Kir_, −20 mV for I_Kd_, and +15 mV for I_Na_). Synapses were blocked with APV, CNQX and bicuculline. Arrowheads show 0 nA (top traces) and −40 mV (bottom traces).

We recorded from 30 hippocampal GCs from the upper blade of the DG from two male and three female mice at either the end of the “day” (ZT0) or the end of the “night” (ZT12) of 12 h light-dark cycle after two weeks of entrainment. Synaptic inputs were all blocked with APV, CNQX and bicuculline (Methods). We did not detect any significant differences between females and males and the data are thus pooled. We also used 2–3 slices per animal during the course of approximately 4–5 hours. To maximize the number of cells recorded we focused on the four distinct ionic currents that can be studied without the need to add pharmacological agents (Fig. 1): *I*_*Kd*_, *I*_*Kir*_, *I*_*Na*_, and *I*_*leak*_.

**Figure 2:**
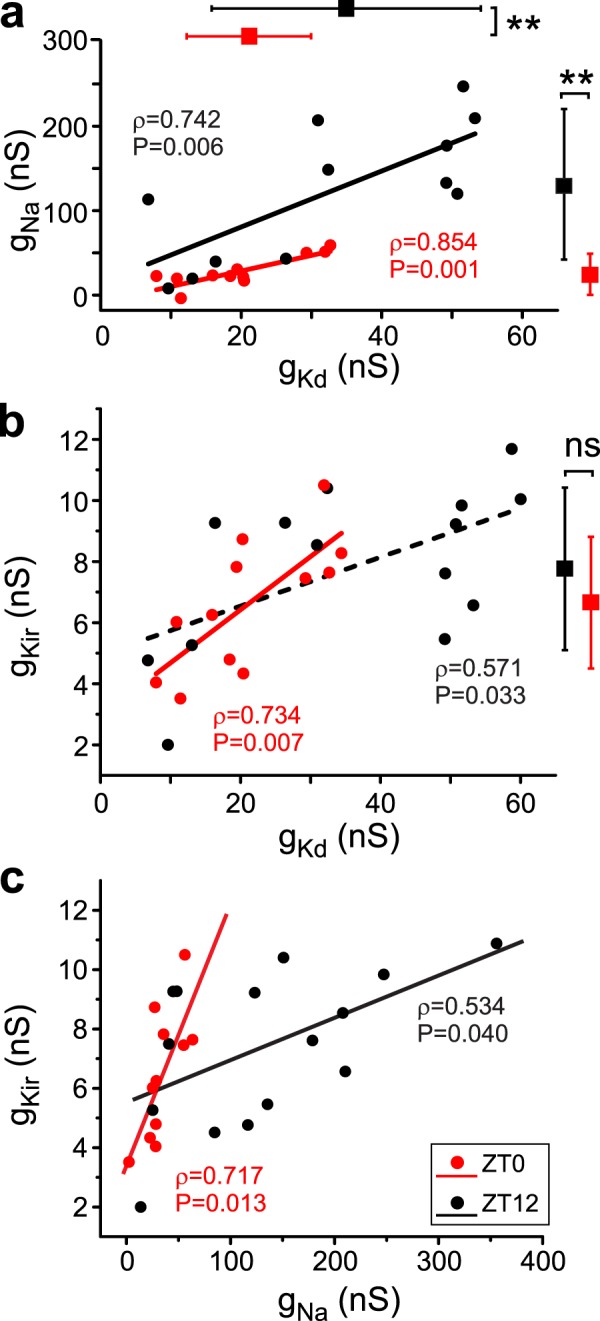
Ionic conductance correlations in hippocampal GCs of 4 month-old mice. g_Kd_, g_Na_, and g_Kir_ are plotted against each other for data recorded at the end-of-day (ZT0, red) and end-of-night (ZT12, black) of a 12 h light-dark cycle. Only these conductance pairs showed significant correlations either at ZT0 or ZT12, shown as Pearson-moment correlation coefficients and their statistical significance. Regression lines are also shown in each panel. The dashed line in b indicates that statistical significance of the correlation is lost after adjustment for multiple comparisons. Means and SD bars for each conductance are shown to the right and top of the plots in a and b. **P<0.001, ns not significant (*t*-Student tests, see Table 1).

**Figure 3:**
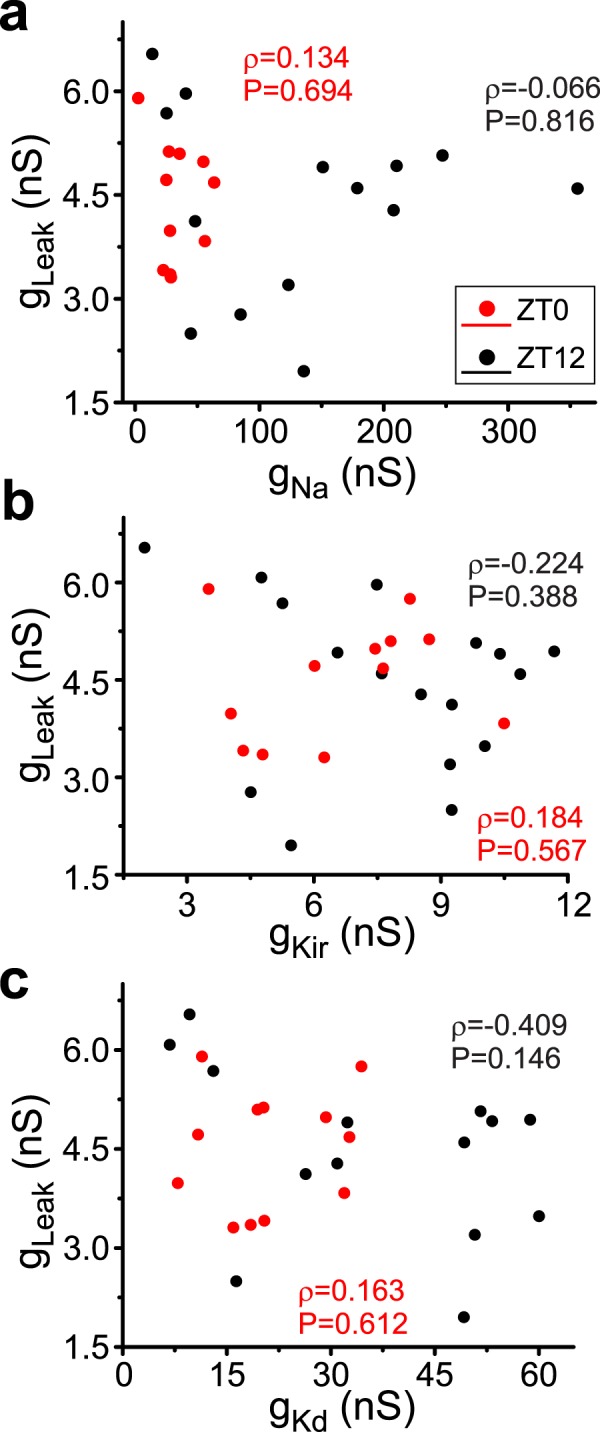
Correlations are not observed when leak conductance in hippocampal GCs is considered: g_Na_, g_Kir_ and g_Kd_ are plotted against *g*_*leak*_ for data recorded at ZT0 (red) and ZT12 (black) of a 12 h light-dark cycle. None of these conductance pairs showed significant correlations either at ZT0 or ZT12, shown as Pearson-moment correlation coefficients and their statistical significance.

As shown in Table 1 and Figs. 2 and 3, the variability of the conductance values of all ionic currents measured both during the day and night was large, with conductance ranges (ratios of max/min) as low as 1.8 for *g*_*leak*_ and as high as 25.9 for *g*_*Na*_, both at ZT12.

**Table 1:** Descriptive statistic of ionic conductances measured in hippocampal GCs.

	ZT0, End-of-day				ZT12, End-of-night			
	*g*_*Kd*_	*g*_*Na*_	*g*_*Kir*_	*g*_*leak*_	*g*_*Kd*_	*g*_*Na*_	*g*_*Kir*_	*g*_*leak*_
Max	60.040	355.850	11.680	6.538	34.410	63.630	10.495	5.900
Min	6.780	13.810	2.000	1.950	7.960	2.460	3.510	3.307
Ratio (max/min)	8.9	25.8	5.8	3.4	4.3	25.9	3.0	1.8
Mean	35.125	132.323	7.808	4.445	21.100	33.955	6.611	4.510
SD	19.074	95.877	2.676	1.310	9.065	17.749	2.148	0.916
n	15	15	17	17	12	11	12	12
*t*-statistic (day vs night)	3.338	3.343	1.283	−0.157				
P	0.0277	0.0014	0.2103	0.8838				
df	25	24	27	27				

Top six rows show descriptive statistical values for the conductances we recorded at ZT0 (left) and ZT12 (right) cycles. The bottom three rows show the nested *t*-Student test comparisons for each conductance between ZT0 and ZT12.

We observe that two of the conductances significantly change average amplitude between ZT0 and ZT12 (*g*_*Kd*_ and *g*_*Na*_) as noted at the bottom of Table 1, and graphically indicated by the means and standard deviation (SD) bars on Fig. 2. Neither *g*_*leak*_ nor *g*_*Kir*_ saw a significant change in mean value (Table 1).

Furthermore, significant linear correlations of conductance levels at ZT0 were noted for the following conductance pairs *g*_*Kd*_*-g*_*Na*_, *g*_*Kd*_*-g*_*Kir*_ and *g*_*Na*_*-g*_*Kir*_ (Fig. 2, red symbols and traces). Pearson product-moment correlations were significant at P <0.05 (shown on each panel and indicated by a solid line) for these pairs, even after adjusting for multiple comparisons (Table 2, left, see Methods). At ZT12 one of these strong relationships (*g*_*Kd*_-*g*_*Kir*_) changes and the significance is lost when multiple comparisons are taken into account (Table 2, right).

**Table 2:** Comparisons of pairwise conductance correlations.

	ZT0			ZT12		
	*g*_*Na*_ *-g*_*Kir*_	*g*_*Kd*_ *-g*_*Kir*_	*g*_*Kd*_ *-g*_*Na*_	*g*_*Na*_ *-g*_*Kir*_	*g*_*Kd*_ *-g*_*Kir*_	*g*_*Kd*_ *-g*_*Na*_
Slope (μS)	0.09	0.17	1.80	0.14	0.08	3.23
R^2^	0.460	0.493	0.699	0.231	0.270	0.511
ρ	0.717	0.734	0.854	0.543	0.571	0.742
P	0.013	0.007	0.001	0.040	0.033	0.006
Adjusted P	0.050	0.025	0.017	0.050	0.025	0.017

Slopes, coefficients of determination (R^2^) and Pearson product-moment correlations (ρ) for the three voltage-gated conductances that show values above ρ > 0.5, and their corresponding P values. Adjusted P values for multiple comparisons of ρ are shown in last row (see Methods). Only *g*_*Kd*_*-g*_*Kir*_ at ZT12 has P > Adjusted P and is therefore not statistically significant.

None of the pairs involving *g*_*leak*_ (*g*_*Kd*_*-g*_*leak*_, *g*_*Na*_*-g*_*leak*_ and *g*_*Kir*_*-g*_*leak*_) showed a significant correlation either at ZT0 or ZT12 (Fig. 3). We take this as a strong indication that the correlations observed only occur between the voltage-gated conductances in these cells (Fig. 3).

Remarkably, we further observed that the slopes of the pairwise conductance correlations described above significantly changed (Welch’s *t*-test) between ZT0 and ZT12 for two of the pairs, *g*_*Kd*_-*g*_*Kir*_ and *g*_*Na*_*-g*_*Kir*_, while the slope of the third pair (*g*_*Kd*_*-g*_*Na*_) remains statistically unmodified (Table 3).

**Table 3:** Comparisons of slopes of pairwise conductance correlations between ZT0 and ZT12.

	g_Kd_-g_Na_	g_Na_-g_Kir_	g_Kd_-g_Kir_
t	1.443	−2.555	−4.689
df	21	21	19
P	0.165	0.018	0.0001
Adjusted P	0.050	0.025*	0.017**

Comparisons of slopes for the three voltage-gated conductances pairs that show Pearson product-moment correlations ρ > 0.5 (slopes and ρ in Table 2) using a Welch’s *t*-test. Adjusted P values for multiple comparisons are shown in last row. *g*_*Na*_*-g*_*Kir*_ and *g*_*Kd*_*-g*_*Kir*_ pairs show statistically significant change in slope between the ZT0 and ZT12 phases (P > Adjusted P).

Altogether these results are a strong indication of the presence of either a circadian-, light/dark- or sleep-dependent regulation of the mean conductances as well as of the conductance correlations between ZT0 and ZT12 of a 12-hour light/dark cycle from among those currents measured in this study. Importantly, neither the mean conductances nor the conductance correlations involving the leak current were affected by the light/dark cycle.

### Basal forebrain ChAT^+^ neurons (BFCs)

**Figure 4:**
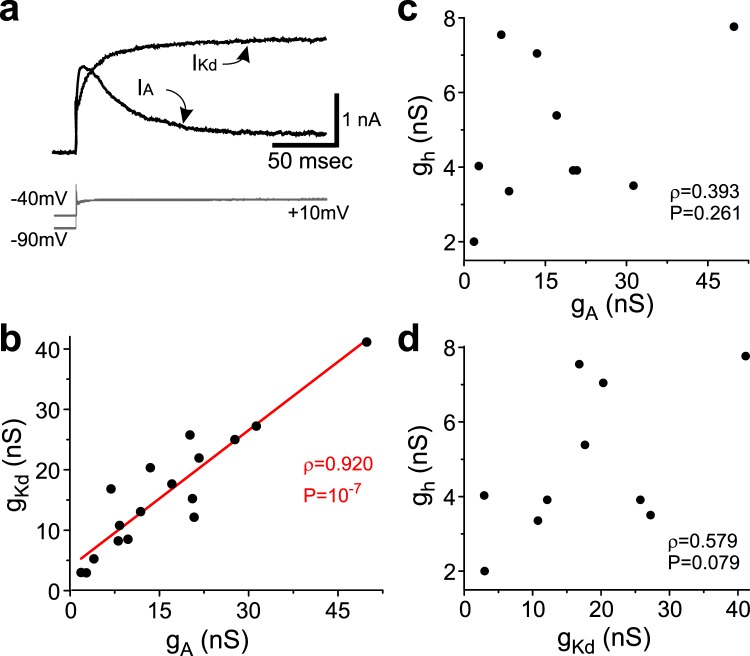
Ionic currents and conductance correlations in adult mouse BFCs. (**a**) Raw leak-subtracted *I*_*Kd*_ and *I*_*A*_ (black traces). Voltage steps used to elicit the currents are shown in gray below the currents. (**b**) *g*_*Kd*_ and *g*_*A*_ of all cells recorded are plotted against each other. (**c**) *g*_*A*_ vs *g*_*h*_. (**d**) *g*_*Kd*_ vs *g*_*h*_. Pearson product-moment correlation coefficients (ρ) and statistical significance (P) are shown in b, c and d; regression line is shown in b.

We recorded from a total of 17 BFCs. We analyzed three ionic currents, *I*_*A*_, *I*_*Kd*_ and *I*_*h*_. *I*_*A*_ and *I*_*Kd*_ were expressed in all 17 cells (see example in Fig. 4a), but *I*_*h*_ was measurable only in 10 of those cells. The range of conductances (ratio of max/min) was broad, similar to hippocampal GCs: 27.0 for *g*_*A*_, 14.0 for *g*_*Kd*_ and 3.9 for *g*_*h*_. However, we detected a statistically significant correlation only for the *g*_*A*_-*g*_*Kd*_ pair (ρ = 0.920, P = 1.7×10^−7^, Fig. 4b). For the remaining two pairs the correlations were not statistically significant: *g*_*A*_-*g*_*h*_ pair (ρ = 0.393, P = 0.261), *g*_*Kd*_-*g*_*h*_ pair (ρ = 0.580, P = 0.079) (Fig. 4c, d).

## Discussion

Correlations of ionic conductance amplitude have been characterized in populations of identical neurons in invertebrates. We set out to test the hypothesis that such correlations are a much more widespread phenomenon expressed in very different animal groups, including two phyla that are widely separated over evolutionary time: chordates (i.e. mammals) and arthropods (i.e. crustaceans). Our analysis on a subset of ionic conductances expressed by two different mouse cell types provides strong evidence for this hypothesis. Moreover, the high incidence of correlations in a subset of conductances (3 out of 4 of the recorded conductances in GCs, and 2 out of 3 in BFCs) suggests that, like in invertebrates, the correlation of ionic conductances is highly prevalent among mammalian neurons. In fact, most neurons, including those reported on here, express a wider variety of currents than those we have measured and it is likely that additional correlations will be observed among these larger sets of currents. In fact, there is no reason to believe the mechanisms governing the generation of these correlations at the biophysical cellular and subcellular levels differ across these species. These correlations probably reflect the existence of common regulatory pathways (see^13,14^) that are important in establishing cell type-specific set-points in conductance space that determine specific neuronal activity attributes (e.g. spiking frequency). These set-points are not immutable, but can shift along trajectories described by the correlation lines, such as those reported here, as neurons respond to persistent stimuli or factors by changing their conductance levels. This would thus enable these neurons to behave in cell-type characteristic ways while allowing the individual currents to vary in amplitude (cf.^25,27^. In fact, it has been suggested that these correlations likely contribute to specifying cell type identity^17,24^.

**Figure 5:**
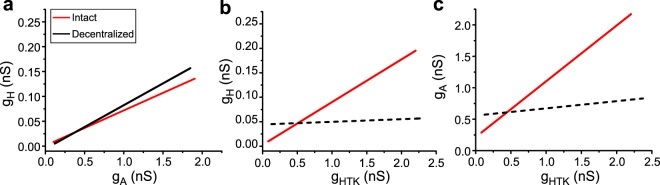
Ionic conductance correlations in PD neurons from adult crab STG. *g*_*A*_ is a transient K^+^ conductance, *g*_*HTK*_ is a high-threshold K^+^ current composed of a delayed rectifier current plus a much larger Ca^++^-dependent current, and *g*_*H*_ is a hyperpolarization-activated monovalent cation inward current. Only the correlation lines (minus symbols) of data originally described in Fig. 2 of Temporal *et al*.^28^ are shown for clarity (please see methods therein). Control data (red lines) were obtained from ganglia fully exposed to the natural neuromodulatory environment of the STG; Decentralized data (black lines) were obtained from ganglia whose neuromodulatory input was removed 24 hrs prior to the recordings. The dashed lines in b and c indicate that the correlations are not statistical significance (P > 0.05) under those conditions.

Ionic conductance correlations reported in several invertebrate preparations^12,15–18^, have been shown to be strongly dependent on neuromodulatory input^15,28^. Figure 5 illustrates this in schematic form for the three conductances described in^28^ for identified PD neurons of the stomatogastric ganglion (STG) neurons of crabs. The individual data have been removed but the correlation lines are as described in Fig. 2 of^28^. We wish to highlight the parallels of the earlier results with those we obtained from GCs and reported here (Fig. 3), in that multiple conductances (in this case, the conductances of the K^+^ currents *g*_*A*_, and *g*_*HTK*_, and those of the inward cationic current *g*_*H*_; see definitions in the legend of Fig. 5 and in Temporal *et al*.^28^) show pairwise correlations (Fig. 5, red traces) when the neuromodulatory environment of the neurons (composed of numerous peptides, amines and other metabotropic agonists^29^) is intact. However, some of these correlations disappear (dashed lines) when neuromodulators are removed (Fig. 5, black traces). Now we have shown evidence that in dentate gyrus GCs the correlations can be regulated in a circadian-like manner. During the light/dark cycle the conductance correlations can either remain unchanged (Fig. 2a), disappear completely (e.g. Fig. 2b) or be significantly modified without completely collapsing (Fig. 2c). This stands in contrast to STG neurons, in which correlations appear to either not change at all (Fig. 5a) or completely disappear (Fig. 5b,c).

This suggests the interesting possibility that multiple set-points along correlation lines that satisfy functional requirements of neurons under certain conditions can themselves be regulated to various degrees, revealed as changes in correlation slopes, under other conditions (e.g. different neuromodulatory or circadian states). The exact contributions of the sleep/wake and circadian cycles to the changes in conductance correlations remain to be determined.

It is interesting that the variances between some of these currents are also subject to a light-dark cycle or circadian regulation in mammalian GCs (Fig. 2) or modulatory state in STG neurons (Fig. 5), sometimes (but not always) concurrent with the changes in correlation slopes. This would indicate that these correlations serve an important functional role that shifts between environmental or internal conditions. The cellular mechanism underlying this regulation is not known. However, a regulation mechanism of ionic correlations has already been shown in crustacean neurons, in which the neuromodulatory environment determines both the existence of conductance correlations^15^ and correlations of RNA levels^28^. Neuromodulators may mediate the circadian-like effects we observe in mouse GCs.

It is also interesting that such conductance correlations appear only to involve voltage-dependent conductances and do not seem to involve the leak conductance of the cells in both crustacean and mammalian neurons. This may reflect that leak conductances are simply defined as the general input conductance of the neurons in our work, and were not characterized on the basis of specific ion channel identities. The existence of more than a single ion channel in the background conductance with different relationships to each other could in principle mask a significant correlation. Alternatively, if conductance correlations serve specific roles in regulating activity attributes, it is possible that voltage-gated channels are best suited for this, and leak channels are too coarse a tool, and thus may not be adequate targets, for correlations.

The most recently proposed mechanism able to generate/control the correlation slopes between conductances involves activity-dependent processes^13,14^. However, we have shown in the past a theoretical accounting of how a combination of both activity (similar to that used by O’Leary *et al*., 2013) and neuromodulatory regulation mechanisms can give rise to homeostatic changes of the rhythmic activity observed experimentally in the pyloric network in crabs^30^. Also, we have recently observed that these correlations are lost in the absence of neuromodulation in around 2 hours (Salloum & Golowasch, unpublished), which seem faster than what would be consistent with an activity-dependent transcription regulation model such as that proposed by O’Leary and collaborators^14^ (although fast ion channel turnover rates of a few hours have been reported^31^). Furthermore, as indicated above, we know from work on crab neurons that correlations are regulated both at the transcription as well as at the translation levels^28^, revealing this a phenomenon that is very complex and likely to be involved in important cellular functions. We think that the ionic correlation generating mechanisms are likely to be qualitatively similar in both vertebrates and invertebrates. More research is required to test this hypothesis.

We conclude that ionic conductance amplitude correlation between subsets of ion currents is a ubiquitous property of neurons across vertebrate as well as invertebrate species, and that they are heavily regulated by multiple mechanisms, likely to be similar across species. This opens the challenge of identifying the mechanisms that co-ordinate the expression of ionic conductances in individual neurons, and give rise to their correlation in populations of identified neurons in different species.

## Methods

We report observations from two different cell types: hippocampal granule cells (GCs) from the upper blade of the dentate gyrus (DG) from 114–128 days (~4 months) old male and female C57BL/6 mice (Jackson Laboratories, Bar Harbor Maine), and choline-acetyl transferase positive neurons from basal forebrain (BFCs) of 30–90 day-old BAC transgenic mice expressing enhanced green fluorescent protein (eGFP) under the promoter of the enzyme choline acetyltransferase, ChAT (B6.Cg-Tg (RP23-268L19-EGFP) 2Mik/J, Jackson Laboratories, Bar Harbor Maine, RRID:IMSR_JAX:007902).

All experiments were performed in accordance with the U.S. Public Health Service Policy on Humane Care and Use of Laboratory Animals, the National Institutes of Health Guidelines for the Care and Use of Animals in Research, and approved by the Rutgers University Institutional Review Board and by the Institutional Animal Care and Use Committee at Johns Hopkins University, where the recordings were performed.

### Hippocampus granule cells (GCs)

C57BL/6 adult (114–116 days old) mice of both sexes were “entrained” for at least 2 weeks in light-tight compartments with 12-hour dark/light cycles. For slice preparation, the mice were removed from their cages 15 minutes before the light-to-dark or dark-to-light transition (scheduled at 10 AM or 2:30 PM). The mice were first deeply anesthetized with isofluorane and then perfused transcardially with cold dissection buffer (5 ml at 10 ml/min) containing 92 mM *N*-methyl-D-glucamine (NMDG), 2.5 mM KCl, 1.25 mM NaH_2_PO_4_, 30 mM NaHCO_3_, 20 mM HEPES, 25 mM glucose, 2 mM thiourea, 5 mM Na-ascorbate, 3 mM Na-pyruvate, 12 mM N-acetyl cysteine, 0.5 mM CaCl_2_ and 10 mM MgSO_4_ pH adjusted to 7.4. After decapitation, brains were removed quickly, and acute hippocampal slices (300 µm) were made as described^32^ in ice-cold dissection buffer bubbled with a mixture of 5% CO_2_ and 95% O_2_. The slices were allowed to recover for 15 min at 30 °C in dissection buffer and then for one hour at room temperature in artificial cerebrospinal fluid (ACSF): 124 mM NaCl, 5 mM KCl, 1.25 mM NaH_2_PO_4_, 26 mM NaHCO_3_, 10 mM dextrose, 1.5 mM MgCl_2_, and 2.5 mM CaCl_2_ bubbled with a mixture of 5% CO_2_ and 95% O_2_.

All recordings were performed in a submerged recording chamber superfused with ACSF (30 ± 0.5 °C, 2 ml/min). Whole-cell voltage-clamp recordings were made from GCs identified with infra-red microscopy and located in the upper blade of the DG away from the tip where the upper and lower blades connect. To minimize patching of immature cells, we focused on cells on the outer layer of the blade away from the hilus. We used borosilicate glass patch pipettes (3–6 MΩ) filled with intracellular solution containing the following: 130 mM K-gluconate, 10 mM KCl, 0.2 mM EGTA, 10 mM HEPES, 4 mM MgATP, 0.5 mM Na_3_GTP, 10 mM Na-phosphocreatine (pH 7.2–7.3, 280–290 mOsm). GCs express a large number of ionic currents^33,34^, but several can be measured without the need of chemical inhibitors. Membrane currents were recorded in the presence of 20 μM 6-cyano-7-nitroquinoxaline-2,3-dione (CNQX), 100 μM 2-amino-5-phosphonovaleric acid (APV) and 10 μM bicuculline methiodide (BMI) to block fast synaptic transmission. Average input resistance was 81.9 ± 21.7 MΩ (range: 64.4 to 115.0 MΩ). Series resistance was <20 MΩ (range 6–20MΩ), and compensation of at least 80% was achieved in every case, which was used to correct for series resistance-induced voltage errors. All drugs were purchased from either Sigma Aldridge (RRID:SCR_008988) or Tocris (RRID:SCR_003689).

### Basal forebrain cells (BFCs)

The B6.Cg-Tg(RP23-268L19-EGFP)2Mik/J mice (RRID:IMSR_JAX:007902) were processed exactly as described in^35^.

### Ionic currents and conductances

In GCs, we measured the following currents: delayed rectifier K^+^ (*I*_*Kd*_), inward rectifier K^+^ current (*I*_*Kir*_), a fast, inactivating and TTX-sensitive inward current (*I*_*Na*_, see below), and the linear leak current (*I*_*leak*_) (Fig. 1). These were all the currents that could be measured without introducing pharmacological agents that may distort the correlations. GCs were clamped both at a holding voltage (V_h_) of either −40 and −90 mV and voltage steps of 500–600 msec duration were applied between −70 and +40 mV in 10 mV increments at 0.33 Hz to measure *I*_*Kd*_, *I*_*Na*_, *I*_*leak*_. For *I*_*Kir*_ we applied 800 msec pulses from V_h_ = −40 mV starting at −120 mV in 5 mV increments. We measured *I*_*Kd*_ (Fig. 1c) after leak subtraction as the current at the end of a step to +40 mV from V_h_ =−40 mV. *I*_*Kir*_ (Fig. 1a,b) was measured after leak subtraction, and *g*_*Kir*_ was calculated as the slope of the leak-subtracted I-V curve between −110 and −90 mV (Fig. 1b). *E*_*Kir*_ was measured as the voltage of this slope line extrapolated at zero current (average End of Day: −72.2 ± 3.4 mV, average End of Night: −71.9 ± 3.2, t = 0.218, df = 27, P = 0.829, t-Student test). *I*_*Na*_ is the early transient inward current we observed in GCs (Fig. 1c) that peaks at around 0 mV (Fig. 1d) and is fully blocked by 1 μM tetrodotoxin (TTX). Finally, *g*_*leak*_ was calculated as the slope of the I-V curve between −60 and −40 mV, which is predominantly a linear component in that voltage range.

In BFCs, we measured the following currents: *I*_*Kd*_, the transient A-type K^+^ current (*I*_*A*_), and the hyperpolarization-activated inward current (*I*_*h*_). These currents could be separated, as in GCs, without the use of pharmacological tools. *I*_*Kd*_ was measured as in GCs. *I*_*A*_ was measured by subtracting the currents obtained from V_h_  = −40 mV from those measured from V_h_ = −90 mV, and *g*_*Kd*_ and *g*_*A*_ were calculated from currents measured at +10 mV, and a drive force using the calculated *E*_*K*_ (−84 mV in hippocampal GCs, −99 mV in BFCs cells). *I*_*h*_ was measured after leak subtraction at the end of a voltage step to −120 mV, and *g*_*h*_ was calculated assuming a reversal potential of −10 mV.

### Experimental Design and Statistical Analysis

Averages are represented as means ± SD and compared with t-Student tests for independent samples. Pearson product-moment correlation coefficients were calculated to reveal correlations between different variables. The Kolmogorov-Smirnov test was used to determine the normality of distributions. These statistical analyses were performed using SigmaStat (Systat Software, Inc., San Jose, CA. USA; RRID:SCR_010285) or Origin (Origin Lab Corp., Northampton, MA, USA; RRID:SCR_015636). Comparison of slopes was performed using a variation of Welch’s *t*-test^36^ as implement in the Microsoft Excel function *SlopesTest*. To adjust for multiple comparisons, we use the false discovery rate method^37^, which essentially increases the stringency of the statistical significance by dividing the chosen ρ = 0.05 by the number of comparisons. Thus, if 4 comparisons are considered, original P values are ranked from higher to lower value, and P for the first comparison only needs to be below ρ = 0.05 to reach significance, while P for the fourth needs to be below ρ = 0.05/4 = 0.0125, and so forth. We refer to this new adjusted ρ as Adjusted P.
